# UCHL1 promotes the proliferation of porcine granulosa cells by stabilizing CCNB1

**DOI:** 10.1186/s40104-024-01043-2

**Published:** 2024-06-11

**Authors:** Shengjie Shi, Huan Yuan, Lutong Zhang, Lei Gao, Lili Zhao, Xiangfang Zeng, Shiyan Qiao, Guiyan Chu, Chuanjiang Cai

**Affiliations:** 1 College of Animal Science and Technology, Northwest A&F University, Yangling, 712100 Shaanxi China; 2 State Key Laboratory of Animal Nutrition, Ministry of Agriculture Feed Industry Center, China Agricultural University, Beijing, 100193 China

**Keywords:** CCNB1, Granulosa cells, Isovitexin, Proliferation, UCHL1

## Abstract

**Background:**

The proliferation of porcine ovarian granulosa cells (GCs) is essential to follicular development and the ubiquitin–proteasome system is necessary for maintaining cell cycle homeostasis. Previous studies found that the deubiquitinase ubiquitin carboxyl-terminal hydrolase 1 (UCHL1) regulates female reproduction, especially in ovarian development. However, the mechanism by which UCHL1 regulates porcine GC proliferation remains unclear.

**Results:**

UCHL1 overexpression promoted GC proliferation, and knockdown had the opposite effect. UCHL1 is directly bound to cyclin B1 (CCNB1), prolonging the half-life of CCNB1 and inhibiting its degradation, thereby promoting GC proliferation. What’s more, a flavonoid compound-isovitexin improved the enzyme activity of UCHL1 and promoted the proliferation of porcine GCs.

**Conclusions:**

UCHL1 promoted the proliferation of porcine GCs by stabilizing CCNB1, and isovitexin enhanced the enzyme activity of UCHL1. These findings reveal the role of UCHL1 and the potential of isovitexin in regulating proliferation and provide insights into identifying molecular markers and nutrients that affect follicle development.

**Supplementary Information:**

The online version contains supplementary material available at 10.1186/s40104-024-01043-2.

## Introduction

Porcine granulosa cells (GCs) play an essential role in follicle development by supplying nutrients [1], secreting hormones [2], supporting and protecting oocytes [3], and regulating the immune system [4]. Primary follicles develop into secondary follicles, then antral follicles, and finally mature follicles; during this process, GCs proliferate from single layers to 2–3 layers and finally to multiple layers to form cavities. Then, GCs proliferate, the follicle cavity expands, and finally, the follicle ovulates [5]. Cell cycle proteins (cyclins) such as CCNB1 and cell cycle-dependent protein kinase (CDKs) regulate cell proliferation [6].

Protein ubiquitination is a process that regulates protein degradation, localization, and function by labeling ubiquitin in cells [7]. Ubiquitination is directly involved in GC proliferation regulation. For example, the E3 ubiquitin ligase anaphase-promoting complex/cyclosome (APC/C) directly regulates cell cycle transition [8]. In addition, ubiquitination and deubiquitination selectively regulate the degradation of essential cell cycle proteins [9]. On the other hand, ubiquitination also regulates the Wnt/β-catenin, NF-κB, and MAPK signaling pathways [10], which affect cell proliferation. Ubiquitin carboxyl-terminal hydrolase 1 (UCHL1, also known as PARK5/PGP9.5) is a deubiquitinating enzyme (DUB) responsible for removing ubiquitin or polyubiquitin from target proteins [11]. *UCHL1* is an essential gene in mouse ovarian follicle development [12]. In particular, our recent study showed that UCHL1 regulates estradiol synthesis in porcine GCs by stabilizing VDAC2, suggesting that UCHL1 plays a deubiquitination role in GCs [13]. What’s more, studies showed that UCHL1 is a cell proliferation marker, particularly in tumor cells [14, 15]. Proliferation and estradiol synthesis are both essential physiological functions of GCs, and they regulate each other, collectively influencing follicle development. However, the role of UCHL1 in GC proliferation remains unclear. Therefore, in this study, on the one hand, we want to explore the role and specific mechanism of UCHL1 in regulating the porcine GC proliferation; on the other hand, we sought to identify upstream small molecules that can activate UCHL1 for use in feeding production practice.

Isovitexin, an active flavonoid substance found in many fruits and vegetables, mediates several biological activities, expressing antioxidant [16] and anti-inflammatory effects [17], and its potential pharmacological activities have been demonstrated [18]. However, regarding cell proliferation, isovitexin has different effects in different cells. Isovitexin can inhibit the proliferation of tumor cells [19]; however, it promotes proliferation of 3T3 fibroblast cells [20]. We found that isovitexin has the potential function of regulating UCHL1 activity by the docking of small molecules with proteins. Nevertheless, the effect of isovitexin on porcine GC proliferation is unknown.

Based on the research background, UCHL1 has been shown to have the potential to regulate porcine GC proliferation. Therefore, we aim to identify the target protein and mechanism through which UCHL1 regulates cell proliferation, and to identify activators of UCHL1 through network pharmacology analysis. Reproductive traits are complex and controlled by multiple genes, making it challenging to identify significant effect genes. However, we have been dedicated to exploring the relevance of UCHL1 in GC function to elucidate its critical role in follicle development. Moreover, screening small molecules that activate UCHL1 to guide production practice.

## Materials and methods

### The culture and transfection of porcine ovarian GCs

Fresh Landrace (180-day-old and 110 kg) ovaries (*n* = 20) were isolated from local slaughterhouses, stored in physiological saline at 37 °C supplemented with 100 IU/mL penicillin (Cytiva, Shanghai, China) and 100 mg/mL streptomycin (Cytiva, Shanghai, China), and shipped to the laboratory within 1 h. The follicular fluid from healthy follicles with a 3–5 mm diameter was removed using a syringe and centrifuged at 1,000 r/min for 10 min at room temperature. The pellet was resuspended in DMEM/F12 (Cytiva, Shanghai, China) containing 10% fetal bovine serum (Gibco, Thermo Fisher Scientific, Shanghai, China), 100 IU/mL penicillin, and 100 µg/mL streptomycin. The cells were incubated at 37 °C in a humidified atmosphere with 5% CO_2_. Twenty ovaries from 10 sows were collected to construct pooled GCs. These GCs were divided into control and experimental groups, and the inoculation density of GCs was 10^5^ cells/cm^2^.

GCs were cultured for 24 h, washed with phosphate-buffered saline (PBS), and the culture medium was replaced. pcDNA3.1-UCHL1 (G0217535-4) (General Biosystems, Anhui, China), sh-UCHL1 (sh0218-1039) (Tsingke Biotechnology, Beijing, China), pcDNA3.1-CCNB1 (G0280294-1) (General Biosystems, Anhui, China) vectors were transfected into GCs at a total of 2,000 ng/well of 6-well plate using X-tremeGENE HP DNA transfection reagent (Roche, Mannheim, Germany). si-CCNB1 (20157) (GenePharma, Suzhou, China) was transfected into GCs at a concentration of 100 nmol/L by X-tremeGENE siRNA transfection reagent (Roche, Mannheim, Germany). The GCs were continued to be cultured in a complete culture medium for 24 h. The sequences of the siRNA and shRNA used are shown in Table 1.

**Table 1:** siRNA and shRNA sequences (pig)

Gene	Sequence (5′→3′)	
	Forward sequence	Reverse sequence
sh-NC	CCGG-GGACTTGCTGCTGCACGAA-CTCGAG-TTCGTGCAGCAGCAAGTCC-TTTTTG	AATT-CAAAAA-GGACTTGCTGCTGCACGAA-CTCGAG-TTCGTGCAGCAGCAAGTCC
sh-UCHL1	CCGG-GGATGGATCGGTTCTGAAA-CTCGAG-TTTCAGAACCGATCCATCC-TTTTTT	AATT-AAAAAA-GGATGGATCGGTTCTGAAA-CTCGAG-TTTCAGAACCGATCCATCC
si-NC	UUCUCCGAACGUGUCACGUTT	ACGUGACACGUUCGGAGAATT
si-CCNB1	GGCUCCUGUACCUGUAUUATT	UAAUACAGGUACAGGAGCCTT

### Quantitative real-time PCR

Total RNA from GCs was extracted using TRIzol reagent (TaKaRa), with the concentration determined on a NanoDrop 2000 spectrophotometer (Thermo Fisher Scientific). Total RNA (500 ng) was reverse-transcribed into cDNA using a Reverse Transcription Kit (TaKaRa, Otsu, Japan). No reverse transcriptase in the reaction system was used as a negative control group. ChamQ SYBR qPCR Master Mix (Q311-02, Vazyme, Nanjing, China) and a StepOne Real-Time PCR instrument (Applied Biosystems, USA) were used for RT-qPCR detection. A two-step PCR reaction procedure was used: predenaturation at 95 °C for 30 s, deformation at 95 °C for 5 s, and annealing at 60 °C for 30 s, a total of 45 cycles. The specificity of each PCR amplification was verified by melting curve analysis. The cDNA template was replaced by pure water as a no-template negative control. The relative mRNA level was normalized to *ACTB* and calculated using the 2^−ΔΔCt^ algorithm. The sequences of the primers used are shown in Table 2.

**Table 2:** Primers used in quantitative real-time PCR (pig)

Gene	Primer sequence (5′→3′)		Accession number
	Forward primer	Reverse primer	
sus-*UCHL1*	ACTGGAGGAGGAGTCTTTGGG	TTCTTGTCCCTTCAGCTCTTCA	NM_213763
sus-*CCNB1*	AATCCCTTCTTGTGGTTA	CTTAGATGTGGCATACTTG	NM_001170768
sus-*CDK1*	CAGCTCGCTACTCAACTCCA	GAGTGCCCAAAGCTCTGAAA	NM_001159304
sus-*CCNE1*	AGAAGGAAAGGGATGCGAAGG	CCAAGGCTGATTGCCACACT	XM_005653265
sus-*CDK4*	AAGTGGTGGGACAGTCAAGC	ACCACCACAGGTGTAAGTGC	NM_001123097
sus-*ACTB*	GATGACGATATTGCTGCGCT	TCGATGGGGTACTTGAGGGT	XM_021086047

### Western blot analysis

The experimental method for Western blot is consistent with the previous report [21]. GCs were washed with PBS and lysed in RIPA lysis buffer (Beyotime Biotechnology, Shanghai, China). Next, 20 µg of total protein was resolved by 12% sodium dodecyl sulfate–polyacrylamide gel electrophoresis (SDS–PAGE) and transferred onto polyvinylidene fluoride (PVDF) membranes (Millipore, Massachusetts, USA). The membranes were blocked (5% skim milk powder dissolved in Tris-buffered saline-Tween) for 2 h at room temperature. The membranes were then incubated with primary antibodies (1:1,000) at 4 °C overnight: anti-UCHL1 (AF5490, Rabbit polyclonal antibody, 25 kDa, Affinity Biosciences, Cincinnati, OH, USA) and (sc-271639, Mouse monoclonal antibody, 25 kDa, Santa Cruz, Dallas, TX, USA), anti-CCNB1 (CY5378, Rabbit monoclonal antibody, 55 kDa, Abways), anti-CDK1 (CY5176, Rabbit monoclonal antibody, 34 kDa, Abways), anti-CCNE1 (CY1028, Rabbit polyclonal antibody, 49 kDa, Abways), anti-CDK4 (CY5836, Rabbit monoclonal antibody, 34 kDa, Abways, Shanghai, China). Subsequently, the membranes were washed three times with Tris-buffered saline-Tween (10 min per time) and stained with secondary antibodies (1:5,000) for 2 h at room temperature: goat anti-rabbit IgG antibodies and goat anti-mouse IgGs (BOSTER, Wuhan, China). Finally, the signals were detected using a gel imaging system (Bio-Rad, CA, USA) and analyzed using Image J software (http://imagej.nih.gov/ij/).

### Flow cytometry

Porcine GCs were cultured in 6-well culture plates at 4 × 10^5^ cells per well. The cells were treated with overexpression plasmids and shRNA for 24 h or 40 mmol/L isovitexin (HY-N0773, Solubility in dimethyl sulfoxide (DMSO): 25 mg/mL, Purity: 99.82%, MCE, New Jersey, USA) dissolved in DMSO for 12 h, then replaced with complete culture medium for 12 h, and then digested with 0.25% trypsin and terminated with DMEM/F12 containing 10% fetal bovine serum. Cells were collected in 70% cold ethanol, fixed overnight at 4 °C, washed twice with PBS, and stained with 50 mg/mL propidium iodide (PI) for 30 min. Finally, the cell cycle status of the GCs was analyzed using flow cytometry (Becton Dickinson, Franklin Lakes, NJ, USA).

### 5-Ethynyl-20-deoxyuridine (EdU) staining

EdU staining was performed according to the manufacturer’s instructions (C10310-1, RiboBio, Guangzhou, China). GCs were seeded in 96-well plates (2 × 10^3^ cells/well) with three repetitions and harvested after treating the cells with overexpression plasmids and shRNA for 24 h or 40 mmol/L isovitexin dissolved in DMSO for 12 h. Cells were stained with 50 mmol/L EdU for 2 h at a final concentration of 50 mmol/L and with Hoechst dye at 25 °C for 15 min after washing three times with PBS. The cells were observed using a Nikon TE2000 microscope (Nikon, Tokyo, Japan), and the data were analyzed using Image J software (http://imagej.nih.gov/ij/).

### Cell counting kit-8 (CCK-8)

GCs were seeded in 96-well plates at 2 × 10^3^ cells per well. After treating the cells with overexpression plasmids and shRNA for 24 h or isovitexin dissolved in DMSO (0, 10, 20, 30, 40 mmol/L) for 12 h, 10 µL CCK8 reagent was added to each well and incubated for 3 h at 37 °C. Finally, the absorbance was measured at 450 nm.

### Co-immunoprecipitation (Co-IP)

For immunoprecipitation analysis, GCs inoculated in three large dishes were gently washed with PBS and lysed with IP cell lysate buffer (C500035, Sangon Biotech, Shanghai, China). 100 μL of supernatant was used for the input group, and the remaining supernatant was added to 10 μL anti-UCHL1/CCNB1/IgG overnight at 4 °C and then was immunoprecipitated with protein G magnetic beads (10003D, Thermo Fisher Scientific, MA, USA) for 2 h. After washing three times with lysate buffer, the magnetic bead-protein complex was finally resuspended in 5 × SDS-PAGE loading buffer and boiled at 95 °C for 10 min. The magnetic beads were then removed and the loading buffer was resuspended with RIPA lysate for immunoblotting.

### In vivo ubiquitylation assays

To detect endogenous CCNB1 ubiquitination levels, GCs were transfected with either shRNA-control or sh-UCHL1 for 24 h. The CCNB1 in the protein supernatants was enriched by immunoprecipitation. Then the treated protein was subjected to a Western blot, which was incubated overnight with an antibody against ubiquitin (Ub) antibody (ab134953, Abcam, Cambridge, UK). The remaining steps were the same as the Western blot. The ubiquitination level of CCNB1 protein was then measured under conditions that interfered with UCHL1.

### Fluorescence microscopy

Porcine GCs were fixed with 4% paraformaldehyde for 20 min, infiltrated with 0.5% Triton-100 for 10 min, blocked with PBS containing 5% bovine serum albumin for 30 min, and incubated with a monoclonal antibody against UCHL1 or CCNB1 at 1:200 for overnight incubation at 4 °C. After washing with PBS three times for 5 min, the cells were incubated with fluorescent secondary antibodies, goat anti-mouse IgG or goat anti-rabbit IgG (Boster, China, Wuhan), for 1 h at 37 °C, and incubated with 4′,6-diamidino-2-phenylindole (DAPI) for 10 min to stain the nucleus. The stained cells are photographed on a Spinning Disk Confocal Microscope (2017165203, Andorra, UK).

### Protein–protein docking, molecular dynamics simulation, and natural molecule-protein docking

The crystal structure of UCHL1 and CCNB1 proteins was searched on the Protein Data Bank (PDB) website (https://www.rcsb.org/), and homologous modeling was performed. Using the Protein–protein docking (Piper) module in Schrodinger, Piper clusters the top 1,000 rotating conformations based on the root mean square deviation (RMSD) between each atom, and the conformation with the highest number of clusters was ranked first. We selected the first conformation for subsequent analysis. We used the Desmond module of the Schrodinger drug design software package to determine the stability of the binding of UCHL1 and CCNB1 proteins. We performed routine molecular dynamics simulations of the conformation of the protein complexes of the above docking proteins.

AlphaFold 2 predicted the protein sequence structure of porcine UCHL1. The active components of Chinese medicinal materials were obtained from the TCMSP database [22]. LeDock was used for molecular docking, and the results were split into several PDB files [23]. The protein structure was imported into Maestro 13.5 for visualization [24]. The interactions between ligand and receptor were analyzed using the interactions Toggle and 2D Sketcher modules of Maestro 13.5.

### Measurement of UCHL1 enzyme activity

The enzyme activity of UCHL1 in GCs was measured using a porcine UCHL1 ELISA Kit (J811272-A, JingMei Biotechnology, Jiangsu, China) according to the manufacturer’s instructions. By measuring the absorbance at a wavelength of 450 nm using a Multiskan TM FC spectrophotometer (Thermo Fisher Scientific, MA, USA), the activity concentration of UCHL1 in the sample can be calculated using a standard curve. The ELISA kit was coated with monoclonal antibodies with no cross-reactions. The detection range is 3–150 U/L. The intra-assay coefficient of variation is less than 10%, and the inter-assay coefficient of variation is less than 15%.

### Statistical analysis

All data are expressed as the mean ± SEM of at least three independent experiments and were performed in triplicate. All analyses were performed using GraphPad Prism 8.0 with Student’s *t*-test, one-way analysis of variance (ANOVA), or two-way ANOVA. Dunnett and Sidak were used for post-hoc tests of one-way and two-way ANOVA tests, respectively. Differences were considered significant at *P < 0.05, **P < 0.01, ***P < 0.001, and ****P < 0.0001.

## Results

### Overexpression of UCHL1 promotes porcine GC proliferation

**Fig. 1:**
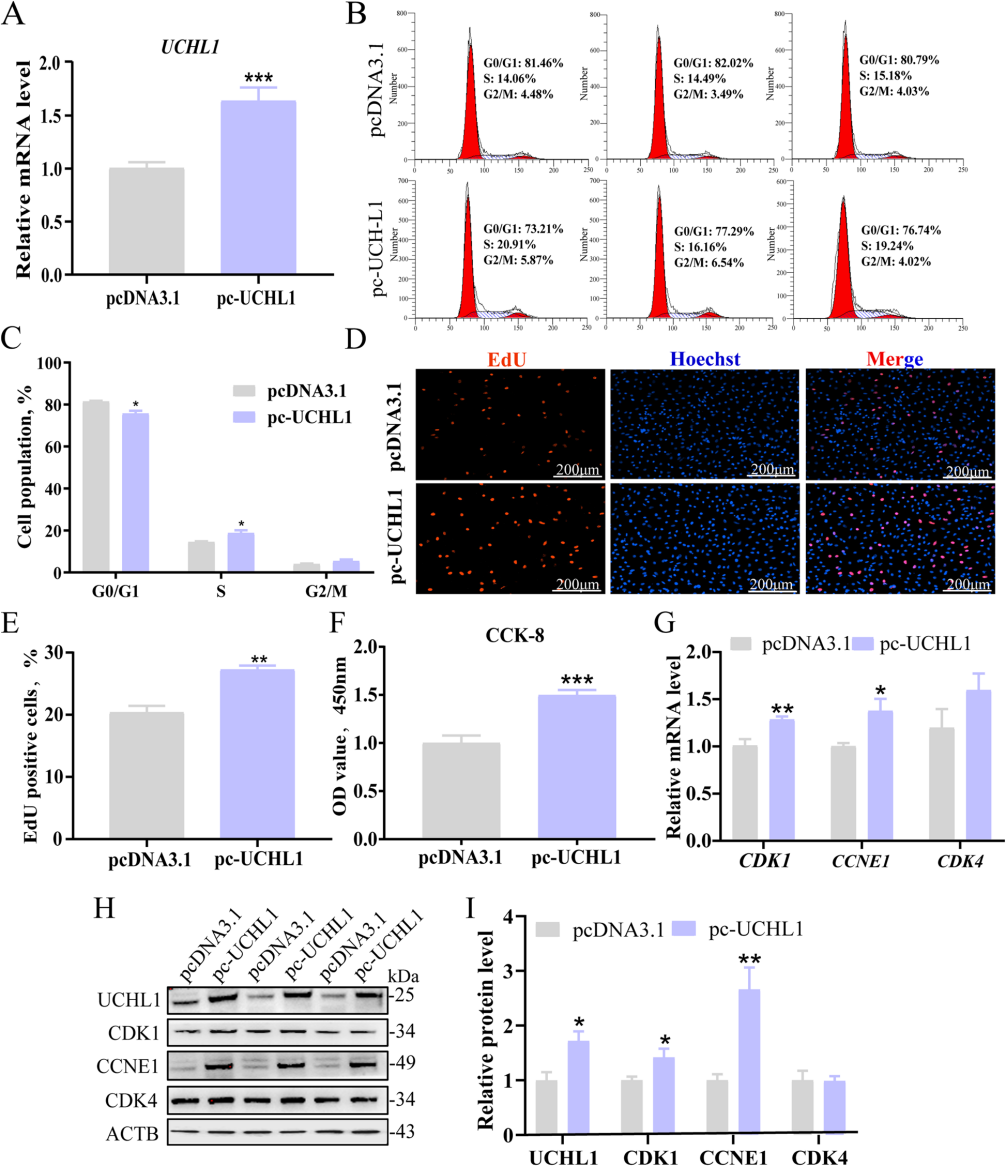
UCHL1 overexpression promotes porcine GC proliferation. **A** The overexpression efficiency of *UCHL1* was detected by RT-qPCR. **B** Cell cycle was detected by flow cytometry at 24 h after transfection of UCHL1 overexpression vector. **C** Cell cycle analysis statistical results. **D** EdU staining assay. GCs in the S-phase were stained with EdU in red, and cell nuclei were dyed with Hoechst in blue. **E** Quantification ratio of EdU-positive cells/total cells. **F** CCK-8 assay detecting cell viability. **G** The mRNA level of cell cycle genes, including *CDK1*, *CCNE1*, and *CDK4* detected by RT-qPCR after overexpression of UCHL1. **H** Western blot analysis of cell cycle proteins. **I** Quantification of protein levels. Data are mean ± SEM of three independent experiments. Significance was determined using Student’s *t*-test, ^*^P < 0.05, ^**^P < 0.01, ^***^P < 0.001

The proliferation of GCs is crucial for follicular development [25]. Importantly, UCHL1 may be significantly involved in this process. To explore the regulatory effects of UCHL1 on GC proliferation, pcDNA3.1 and pc-UCHL1 were evaluated. The mRNA level (Fig. 1A) and the protein level (Fig. 1H and I) of UCHL1 in GCs were significantly increased after transfection with pc-UCHL1. Flow cytometry results showed that after UCHL1 overexpression, the proportion of GCs in the S phase increased, while the proportion of cells in the G1 phase decreased (Fig. 1B and C). Moreover, treatment with pc-UCHL1 increased the number of EdU-positive cells (Fig. 1D and E), and cell viability (Fig. 1F). The mRNA (Fig. 1G) and protein levels (Fig. 1H and I) of cell cycle-related genes (CDK1 and CCNE1) were also increased significantly.

### Interference with UCHL1 inhibits porcine GC proliferation

**Fig. 2:**
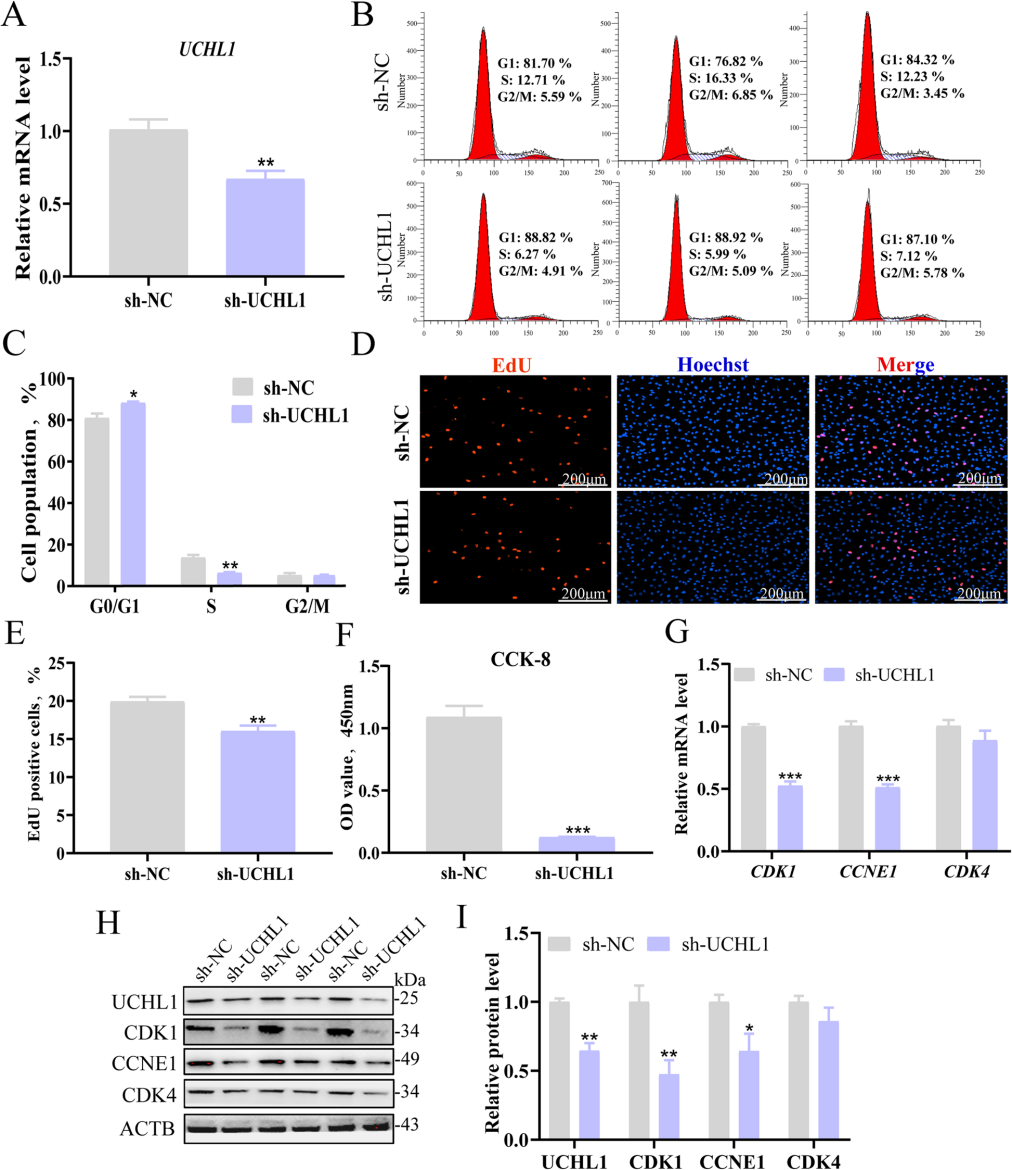
Knockdown UCHL1 inhibits porcine GC proliferation. **A** The inhibitory efficiency of *UCHL1* was detected by RT-qPCR after transfection with sh-UCHL1 compared with NC at 24 h. **B** Cell cycle was detected by flow cytometry at 24 h after transfection of UCHL1 inhibitory vector. **C** Cell cycle analysis statistical results. **D** EdU staining assay. GCs in the s-phase were stained with EdU in red, and cell nuclei were dyed with Hoechst in blue. **E** Results presented as red/blue cell nuclei. **F** CCK-8 assay detecting cell viability. **G** The mRNA levels of cell cycle genes, including *CDK1*, *CCNE1*, and *CDK4* were detected by RT-qPCR after the inhibitory of UCHL1. **H** Western blot analysis of cell cycle proteins. **I** Quantification of protein levels. Data are mean ± SEM of three independent experiments. Significance was determined using Student’s *t*-test, ^*^P < 0.05, ^**^P < 0.01, ^***^P < 0.001

We constructed a UCHL1 interference vector using a shRNA plasmid to investigate the effect on GC proliferation. The mRNA (Fig. 2A) and protein levels (Fig. 2H and I) of UCHL1 were significantly reduced after transfection of the sh-UCHL1 plasmid. Then, the distribution of the cell cycle in GCs was detected using flow cytometry. As expected, sh-UCHL1 significantly decreased the number of cells in the S phase and increased the number of cells in the G1 phase (Fig. 2B and C). The EdU staining assay showed fewer EdU-labeled cells in UCHL1 suppression-treated cells (Fig. 2D and E). The CCK-8 assay revealed that interference with UCHL1 significantly decreased viability (Fig. 2F). In addition, sh-UCHL1 markedly reduced mRNA and protein levels of CDK1 and CCNE1 (Fig. 2G–I).

### Validation of CCNB1 as a deubiquitinase target for UCHL1 in porcine GCs

**Fig. 3:**
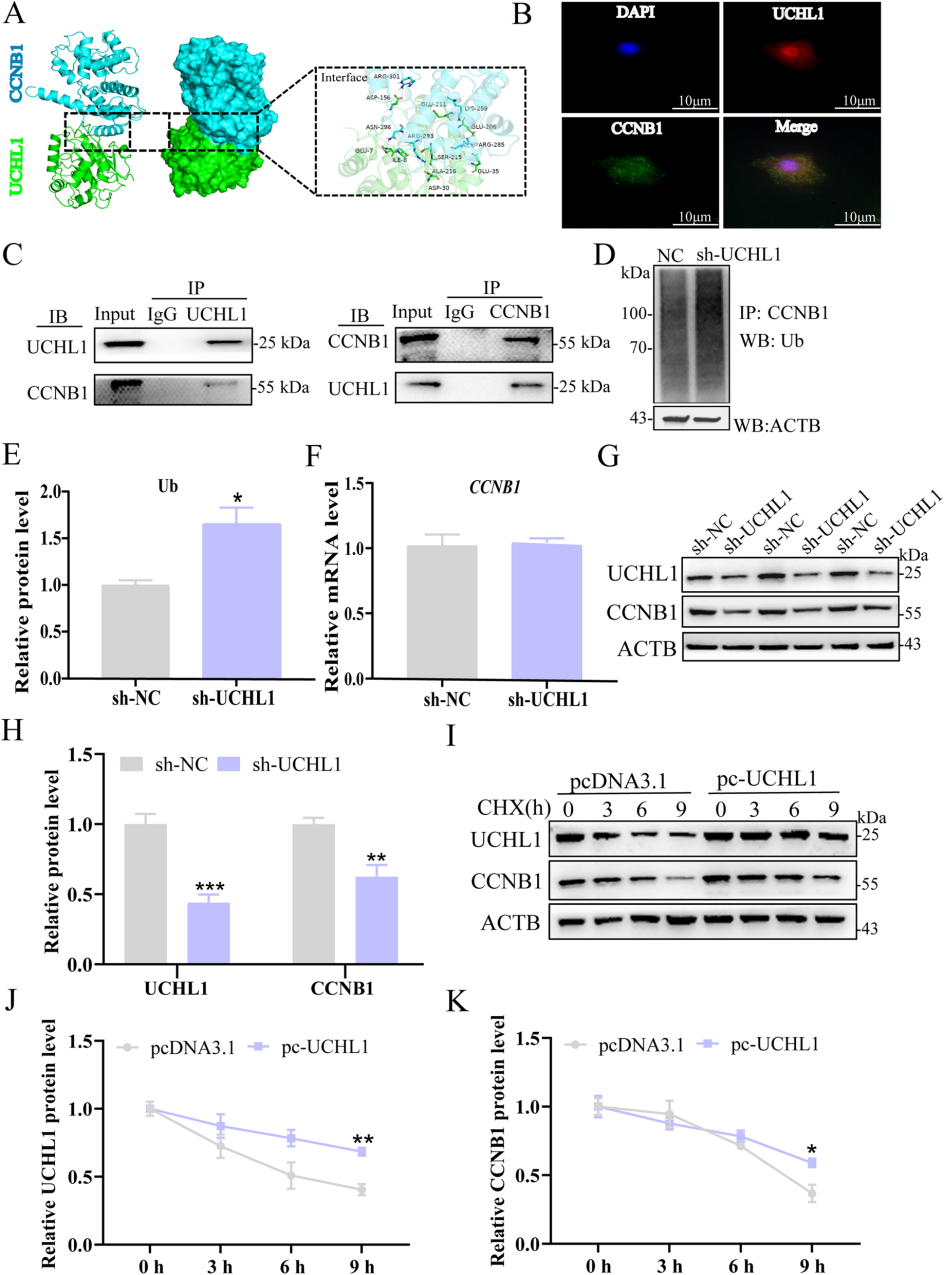
Identification of CCNB1 as a direct target of UCHL1. **A** Molecular docking conformation diagram of UCHL1 and CCNB1. **B** Confocal immunofluorescence staining was used to detect the localization of UCHL1 and CCNB1 in GCs. **C** Co-IP assay was used to reveal an interaction between UCHL1 and CCNB1. **D** Immunoprecipitation of protein extracts was performed using an anti-CCNB1 antibody, followed by immunoblot analysis using an anti-ubiquitin antibody. **E** Quantification of the Ub protein level after treatment with (**D**). **F** Relative mRNA level of *CCNB1* in GCs after knocking down UCHL1. **G** Protein levels of UCHL1 and CCNB1 were detected using Western blot analysis. **H** Western blotting quantitative analysis of UCHL1 and CCNB1. **I** The protein levels of UCHL1 and CCNB1 were detected after treating GCs with pcDNA3.1 or UCHL1 overexpression, followed by treatment with 25 µg/mL cycloheximide (CHX) for the indicated times. **J** and **K** Quantifying the Western blot analysis of UCHL1 and CCNB1 under (**I**) treatment. Data are mean ± SEM of three independent experiments. Significance was determined using Student’s *t*-test, ^*^P < 0.05, ^**^P < 0.01, ^***^P < 0.001

It has been shown that UCHL1 stabilized the expression level of CCNB1 in uterine serous carcinoma [15], and *CCNB1* is a critical gene in cell cycle regulation. However, in porcine GCs, it is also worth clarifying. Protein–protein docking showed that UCHL1 formed a stable binding structure with CCNB1 (Fig. 3A). UCHL1 and CCNB1 were co-localized in GCs by confocal assay (Fig. 3B). The Co-IP assay also demonstrated that UCHL1 and CCNB1 can pull down one another (Fig. 3C). After knocking down UCHL1, the polyubiquitination level of CCNB1 was significantly increased (Fig. 3D and E). The protein level was significantly decreased (Fig. 3G and H). Still, the mRNA of *CCNB1* was not affected (Fig. 3F). Furthermore, the half-life of CCNB1 was prolonged after UCHL1 overexpression compared with the control group treated with the protein synthesis inhibitor cycloheximide (CHX) (Fig. 3I–K). These results demonstrate that UCHL1 enhances the stability of CCNB1 protein level by deubiquitination.

### CCNB1 mediated the promoting effect of UCHL1 on GC proliferation

**Fig. 4:**
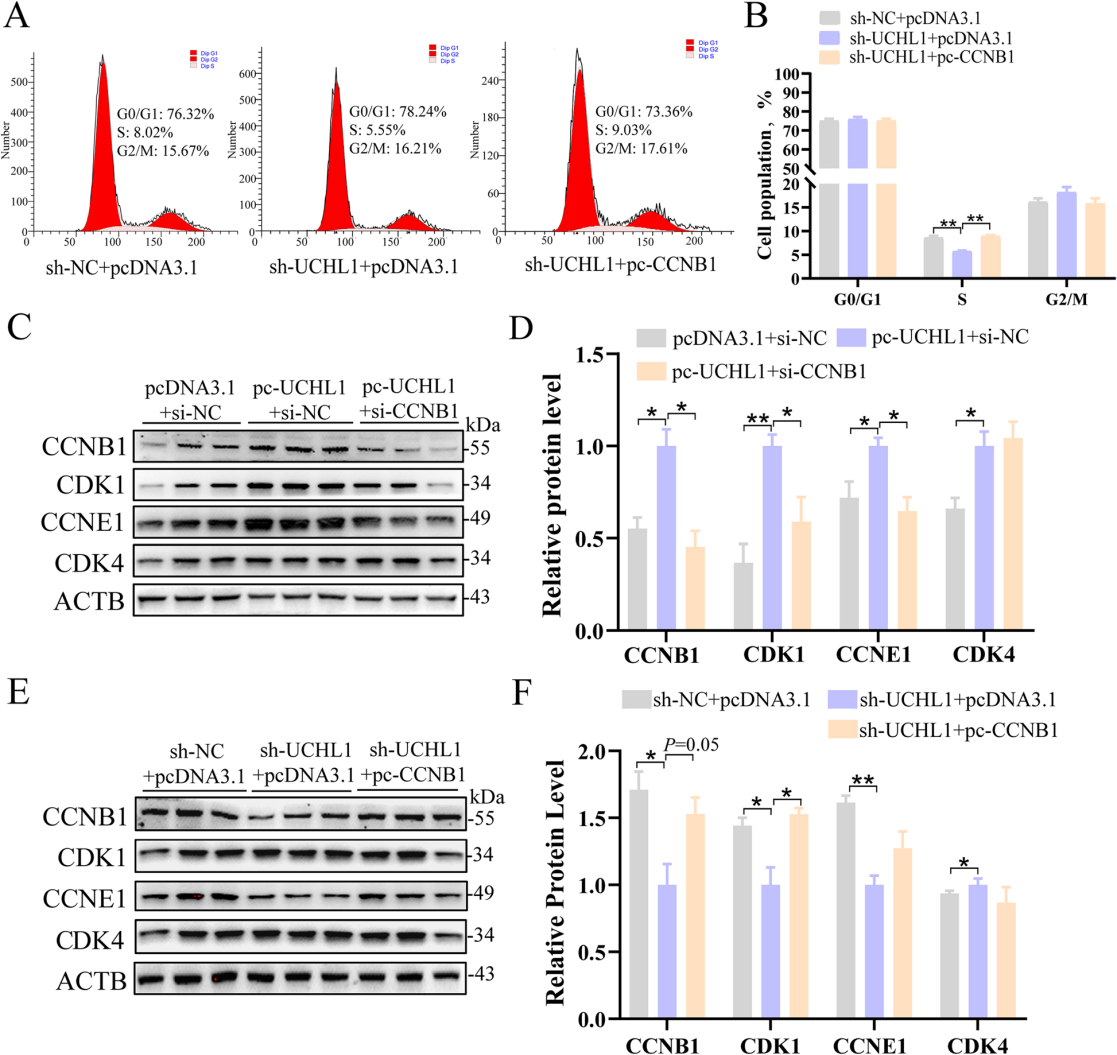
CCNB1 mediated the promoting effect of UCHL1 on proliferation in GCs. **A** Cell cycle was detected by flow cytometry at 24 h after sh-UCHL1 co-transfection with pc-CCNB1. **B** The statistical results of flow cytometry. **C** and **E** Representative protein levels of cell cycle proteins (CCNB1, CDK1, CCNE1, and CDK4) after pc-UCHL1 co-transfection with si-CCNB1 (**C**) and after sh-UCHL1 co-transfection with pc-CCNB1 (**E**). **D** and **F** Quantifying the Western blot analysis under (**C**) and (**E**) treatment. Data are mean ± SEM of three independent experiments. Significance was determined using one-way ANOVA, ^*^P < 0.05, ^**^P < 0.01

We conducted a series of recovery tests to clarify the role of CCNB1 in UCHL1 promoting GC proliferation. The flow cytometry results showed that the proliferation of GCs with UCHL1 knockdown decreased, but the number of GCs in the proliferative stage increased with overexpression of CCNB1 under the condition of knocking down UCHL1 (Fig. 4A and B). As expected, protein levels of cyclins (CCNB1, CDK1, CCNE1, and CDK4) were significantly higher in the co-treated group than those in the interference UCHL1 group (Fig. 4E and F). Similarly, when knocking down CCNB1 in the presence of UCHL1 overexpression, the protein levels of cyclins (CCNB1, CDK1, CCNE1, and CDK4) were significantly lower (Fig. 4C and D). These findings suggest that CCNB1 mediates UCHL1 promotion of GC proliferation.

### Isovitexin promotes UCHL1 enzyme activity and GC proliferation

**Fig. 5:**
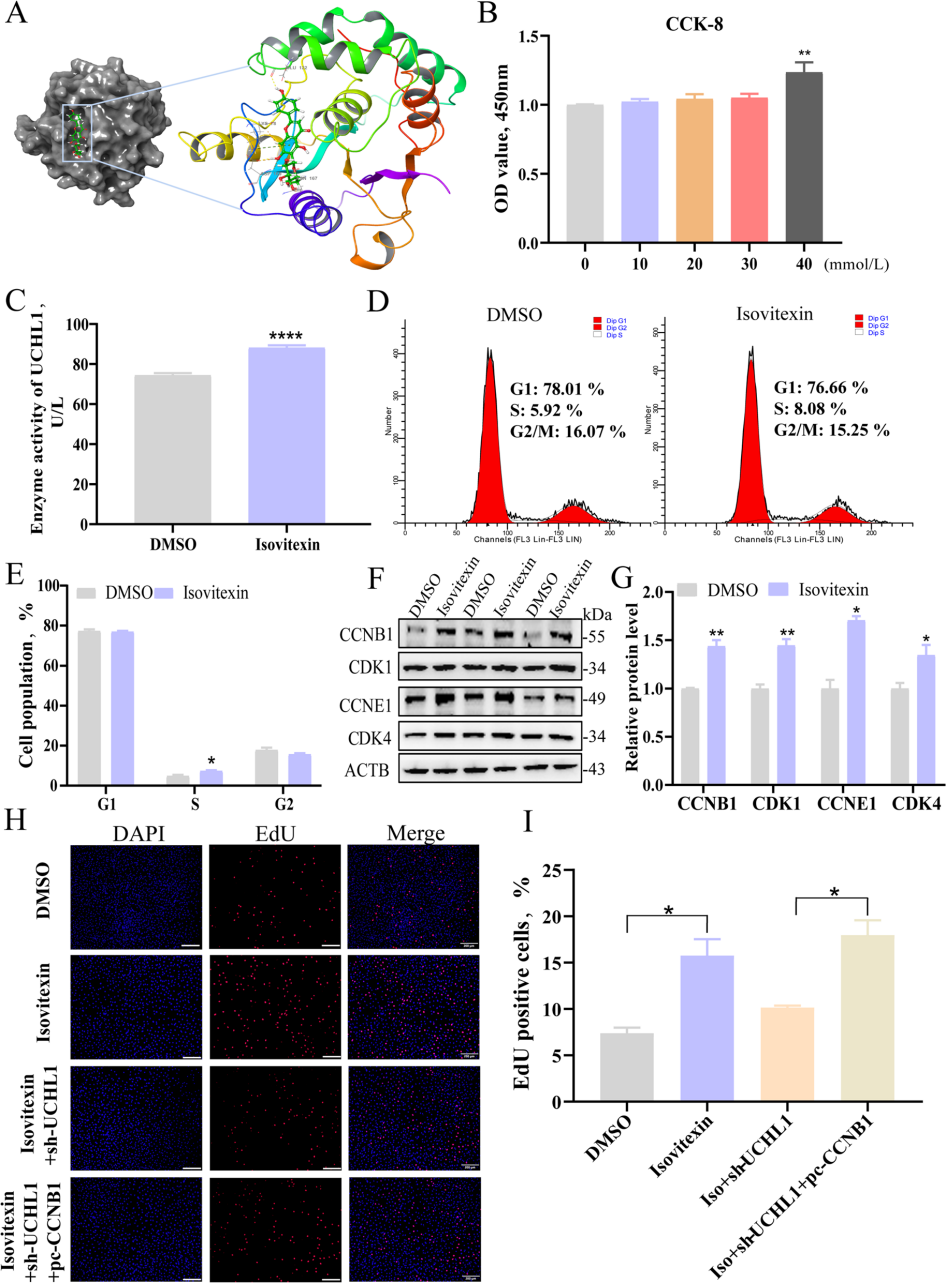
Isovitexin promotes UCHL1 enzyme activity and promotes GC proliferation. **A** Molecular docking conformation diagram of isovitexin and UCHL1. **B** The cell viability of GCs treated with various concentrations (0, 10, 20, 30, 40 mmol/L) of isovitexin was measured using CCK8. **C** ELISA assay of UCHL1 enzyme activity after treating GCs 12 h with 40 mmol/L isovitexin. **D** Cell cycle was detected by flow cytometry at 24 h after treating GCs 12 h with 40 mmol/L isovitexin. **E** Cell cycle analysis statistical results. **F** Western blot analysis of cell cycle proteins. **G** Quantifying the Western blot analysis. **H** EdU staining assay. GCs in the s-phase were stained with EdU in red, and cell nuclei were dyed with Hoechst in blue after DMSO, isovitexin, isovitexin and sh-UCHL1, and isovitexin, sh-UCHL1 and pc-CCNB1 different treatments. **I** Results presented as red/blue cell nuclei. Data are expressed as mean ± SEM of three independent experiments. Significance was determined using Student’s *t*-test and two-way ANOVA, ^*^P < 0.05, ^**^P < 0.01, ^****^P < 0.0001

To make this study more meaningful for animal production, we identified the natural active small molecule substances interacting with UCHL1 in the TCMSP database (Additional file 1), among which isovitexin binds stably to UCHL1 in molecular conformation (Fig. 5A). When isovitexin dissolved in DMSO was added to the medium, 40 mmol/L promoted cell viability (Fig. 5B), UCHL1 enzyme activity (Fig. 5C) and proliferation ability (Fig. 5D and E). Isovitexin also elevated cyclin protein levels in GCs (Fig. 5F and G). The EdU assay showed that the number of GCs in the proliferation phase increased after isovitexin treatment. However, co-treatment of adding isovitexin and knocking down UCHL1 decreased the number of GCs in the proliferation phase. Subsequently, when CCNB1 was reintroduced, the cell count recovered (Fig. 5H and I).

## Discussion

Ubiquitination regulates physiological and pathological processes and conditions in reproduction [26], such as endometriosis [27], spermatogenesis [28], and ovarian folliculogenesis [29]. UCHL1 is a crucial deubiquitination enzyme that has also been studied heavily in several cell types [29, 30]. UCHL1 promotes oocyte maturation, embryo development [31], and endometrial development [32]. In particular, in 2017, *UCHL1* was identified as a candidate gene affecting litter sizes in Chinese Erhualian pigs [33], followed by experiments in 2022 confirmed that the UCHL1-deficient mouse line showed stunted ovarian development and reduced litter size [12]. These findings shed fresh light on UCHL1 as a potential influence factor on ovarian development and have aroused wide attention. In addition, ovary and follicular development rely on GC proliferation [34]. Nevertheless, the effects and mechanisms of UCHL1 on GC proliferation are unknown. In the present study, UCHL1 promoted the proliferation of porcine GCs, which is consistent with the role of UCHL1 in many other cells [15, 35, 36]. Furthermore, UCHL1 markedly upregulated the expression levels of cyclins. Then, what is the relationship between UCHL1 and essential cyclins? Identifying UCHL1 as a regulator of GC proliferation suggests that further investigation into the target proteins influenced by UCHL1 is warranted.

The proliferation process of GCs involves the intricate regulation by several cyclins in a complex network [37]. CCNB1 expression increases in the S phase, reaches the highest level in the G2 and M phases, and enters the nucleus to form complexes with CDK1. CCNE1 controls the G1/S transition of GCs [38]. UCHL1 overexpression significantly increased CCNE1 levels, while the proportion of cells in the S phase and the level of CDK1 significantly increased; however, there was no change in CDK4 levels. This finding suggests that UCHL1 may affect proteins controlling the entry of cells into the S or M phase. In uterine serous cancer cells, UCHL1 deubiquitinates CCNB1 directly to make it more stable [15], as in B cell lymphomas [36]. Consistent with this, we found that UCHL1 co-localizes with CCNB1 in porcine GCs and enhances the stability of CCNB1 by prolonging its half-life through deubiquitination. Nevertheless, the specific mechanisms by which UCHL1 removes ubiquitin chain types and lysine sites from CCNB1 remain unknown.

CCNB1 plays a role in the cell cycle transition from the G2 phase to the M phase [39, 40], still, the flow cytometry results demonstrated that after overexpression of UCHL1, the proportion of cells in the S phase increased significantly, but the proportion of cells in G2/M did not change. There are several reasons for this phenomenon. On the one hand, the cell cycle is a complex and dynamic biological process [41]. CCNE1, which controls the entry of cells into the S phase, undergoes notable changes. It may be that the cells perceive that the M phase is prolonged due to the enhanced stability of CCNB1, and more cells are required to enter the division phase to promote cell proliferation. Therefore, it mobilizes more CCNE1 to accelerate DNA replication. On the other hand, flow cytometry could not distinguish between G2 and M stage cells, and it is possible that the cells rapidly changed from G2 to M stage, resulting in the shortening of the G2 stage and the prolongation of the M stage; however, the number of cells detected in G2/M did not change remarkably. It is common for CCNB1 expression level to change significantly but not affect the G2/M cell ratio [21, 42].

Although our study clarified the role and mechanism of UCHL1 in promoting GC proliferation by stabilizing CCNB1, molecular breeding involves long-term selection and continuous improvement, and nutritional regulation can quickly adjust production performance. Therefore, we are committed to identifying small molecules that can affect the enzyme activity of UCHL1 to achieve rapid and precise regulation of porcine follicle development in a binding manner. In the TCMSP database, we found that isovitexin, an active flavonoid substance, binds stably to UCHL1; this finding attracted our attention because of its wide range of sources [43] and low price. In human disease models, isovitexin has been widely studied due to its anti-inflammatory and antioxidant effects [44]. However, the effects of different flavonoids on cell function are inconsistent; for example, quercetin inhibits steroidogenesis and angiogenesis of porcine GCs [45], and high doses of grape seed extract and proanthocyanidin B2 inhibit the proliferation of human primary luteinized granulosa cells (hGC) and tumor granulosa cell lines (KGN) [46]. However, there are few reports about the effect of isovitexin on GC proliferation. Isovitexin facilitated the proliferation of synovial cells [47] and 3T3 fibroblast cells [20], which is consistent with our findings. However, these studies need to determine mechanisms. Our results suggest that isovitexin promotes GC proliferation by enhancing the enzyme activity of UCHL1; this finding may provide insights into the molecular mechanism by which isovitexin regulates cell proliferation. Nevertheless, the specific mechanism by which isovitexin enhances UCHL1 enzyme activity remains to be further studied.

Based on the research progress on UCHL1 in GC function, we suppose *UCHL1* may be a potential major gene affecting follicular development. The next step of the study will involve genotyping UCHL1 in a large population of pigs and conducting an association analysis with reproductive traits. This aims to provide candidate gene markers and evidence for pigs’ genetic improvement and breeding progress. However, molecular breeding is a prolonged process. Building upon verifying isovitexin’s effects on GC proliferation and regulation of UCHL1 activity at the cellular level, we aim to conduct feeding practice with isovitexin supplementation to validate its impact on reproductive performance in pig herds during actual production. These will be the ongoing objectives of the study.

## Conclusions

**Fig.6:**
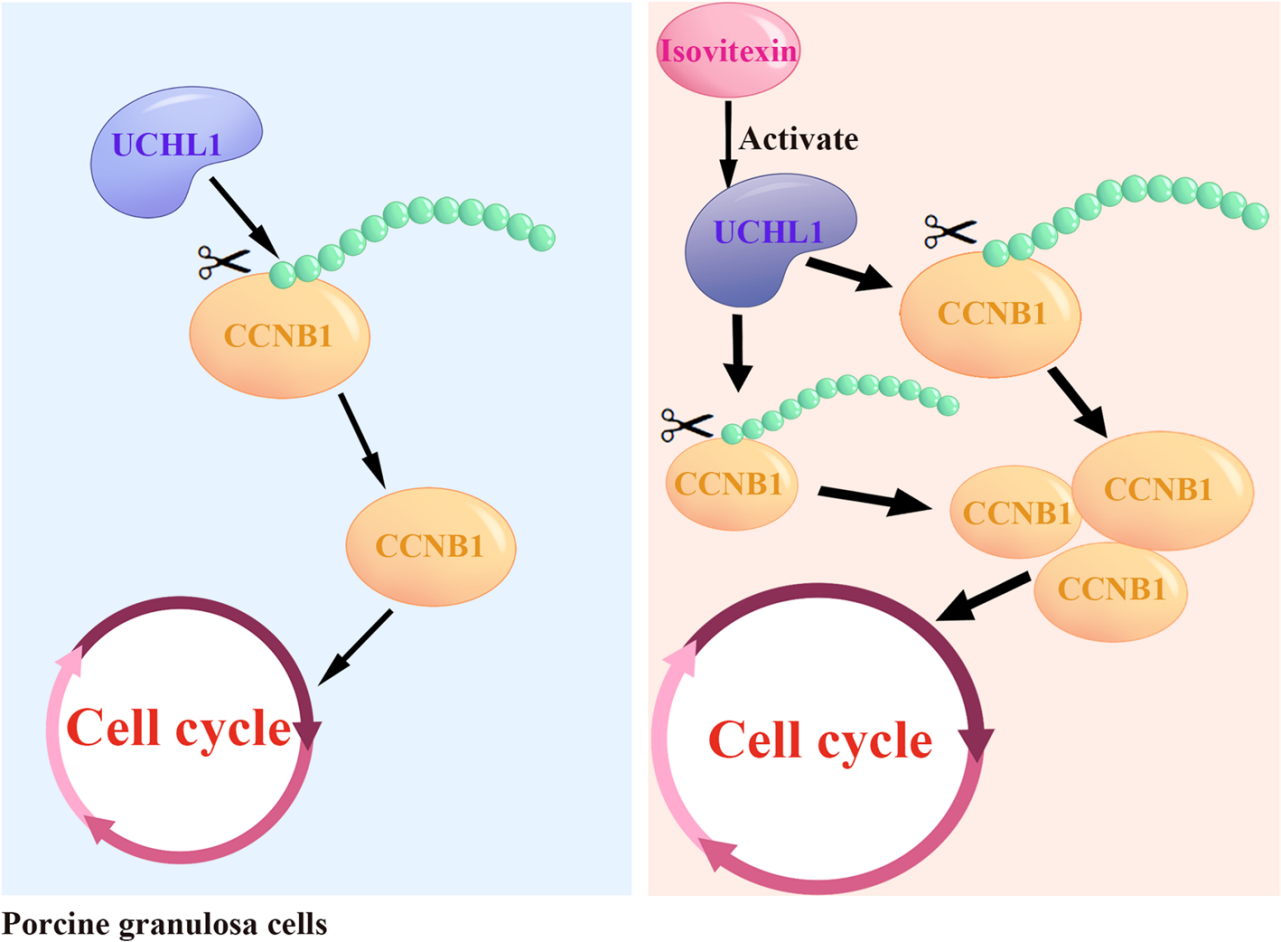
Schematic summary of isovitexin regulating GC proliferation through UCHL1. Isovitexin promotes GC proliferation by stabilizing CCNB1 after increasing the enzyme activity efficiency of UCHL1

In summary, we determined a function of UCHL1 in porcine GC proliferation and identified the flavonoid isovitexin, which enhances its enzyme activity. Isovitexin promotes GC proliferation by stabilizing CCNB1 after activating UCHL1 (Fig. 6). These findings contribute to the understanding of the role of UCHL1 and isovitexin in regulating the GC proliferation and provide new insights into screening molecular markers and nutrients that affect follicle development.

### Supplementary Information

**Additional file 1.** Natural compounds that bind to UCHL1.

## Data Availability

The data sets used and analyzed during the current study are available from the corresponding author on reasonable request.

## Abbreviations

CCNB1: Cyclin B1
CCNE1: Cyclin E1
CDK1: Cyclin-dependent kinase 1
CDK4: Cyclin-dependent kinase 4
CHX: Cycloheximide
DUBs: Deubiquitinating enzymes
ELISA: Enzyme-linked immunosorbent assay
GC: Granulosa cell
IP: Immunoprecipitation
shRNA: Short hairpin RNA
UCHL1: Ubiquitin carboxyl-terminal hydrolase 1
